# Flavivirus Nonstructural Protein 1‑Driven Coagulation via Tissue Factor-Bearing Microvesicles: A Pilot Study

**DOI:** 10.1021/acsomega.5c09129

**Published:** 2025-11-14

**Authors:** Silvia Beltrami, Matteo Ferraresi, Giorgia Cianci, Marco Narducci, Roberta Rizzo, Marcello Baroni, Daria Bortolotti

**Affiliations:** † Department of Environmental and Prevention Sciences, 9299 University of Ferrara, Ferrara 44121, Italy; ‡ 83908 Temple University, Japan Campus, Tokyo 154-0004, Japan; § Department of Life Sciences and Biotechnology, 9299 University of Ferrara, Ferrara 44121, Italy

## Abstract

Flaviviruses, such as dengue and West Nile, cause diverse clinical symptoms and trigger varied clinical outcomes, partly driven by the secreted nonstructural sNS1 protein (sNS1). This study examined how sNS1 from neurotropic and hemorrhagic flaviviruses affects coagulation and inflammation using human monocytic (THP-1) cells. sNS1 from neurotropic viruses (e.g., West Nile, Japanese encephalitis) increased tissue factor (TF) expression and the release of TF-bearing microvesicles, promoting procoagulant activity. In contrast, sNS1 from hemorrhagic viruses (e.g., dengue) showed anticoagulant effects. These divergent responses correlated with the virus-specific modulation of interleukin-6 (IL-6), suggesting that inflammation plays a central role in sNS1-mediated vascular changes. The findings identify sNS1 as a key virulence factor influencing flavivirus pathogenesis through hemostatic and immune pathways, offering a potential target for therapeutic intervention.

## Graphical abstract

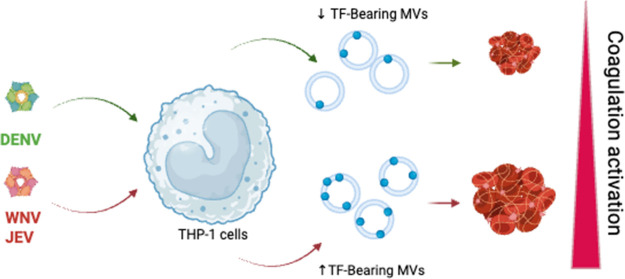

## 1 Introduction

Flaviviruses, genus *Orthoflavivirus*, include several significant arthropod-borne viruses, such as dengue virus (DENV), West Nile virus (WNV), and yellow fever virus (YFV). Collectively, these viruses give rise to clinical manifestations that range from mild self-limited febrile illnesses to more life-threatening sequelae, including hemorrhagic fever,^1,2^ encephalitis,^3^ and multiorgan failure.^4^ One prominent feature of severe flavivirus infections, particularly DENV, is the disruption of hemostasis, leading to vascular leakage, coagulopathy, and, in some cases, hemorrhagic manifestations.^5^ Despite similar genomic structures and replication strategies, flaviviruses exhibit distinct pathogenetic effects, including differential impacts on coagulation and vascular stability.

The nonstructural protein 1 (NS1) protein is a central actor in flavivirus disease mechanisms,^6^ and its significance in virus-driven hemostatic disorders is now clearly established.^7^ NS1 is a multifunctional glycoprotein involved in viral replication, immune modulation, and vascular pathology,^6,8^ characterized by a high degree of sequence homology across the different flaviviruses, with a predominance of fully conserved residues, suggesting that NS1 plays a crucial structural and functional role across the flavivirus family.^9^ It is secreted in high quantities during infection following two main models of oligomerization process, which leads to the formation of tetramers and hexamers, which vary among flaviviruses.^10^ Secreted soluble (s)­NS1 oligomers interact with endothelial cells and disrupt the integrity of the vascular barrier,^11^ leading to increased permeability. This interaction may influence host coagulation and fibrinolysis pathways, triggering either procoagulant or anticoagulant effects, depending on the infecting virus.

Indeed, flavivirus infections have been linked to significant changes in the coagulation profile. One of the hallmark features is the activation of the coagulation cascade, leading to a procoagulant state characterized by increased tissue factor (TF) expression and thrombin generation. This is accompanied by the consumption of clotting factors and platelets, precipitating thrombocytopenia and, in the more severe cases, disseminated intravascular coagulation (DIC). In severe cases, such as dengue hemorrhagic fever or dengue shock syndrome, these abnormalities can lead to vascular leakage, hemorrhagic manifestations, and organ dysfunction. For example, DENV sNS1 has been shown to activate platelets and complement pathways, enhancing coagulopathy and vascular leakage,^12^ while WNV sNS1 appears to predominantly induce endothelial dysfunction, primarily at the central nervous system level,^13^ without significant hemorrhagic manifestations.^14^

Furthermore, dysregulation of the fibrinolytic system and excessive inflammatory responses exacerbate endothelial damage and coagulation imbalance. These findings underscore the complex interplay between viral factors, such as sNS1, and the host’s hemostatic and immune systems during flavivirus infections.^7^

In addition to sNS1, host factors—including the IL-6 response—now emerge as central determinants of endothelial and coagulation balance during viral infections. IL-6, a pro-inflammatory cytokine, is a key mediator in the immune response and exerts significant effects on vascular function and coagulation pathways.^15,16^ Elevated IL-6 levels during flavivirus infections have been implicated in endothelial activation, promoting the expression of adhesion molecules and the recruitment of immune cells, which may exacerbate vascular leakage. IL-6 also drives the production of acute-phase proteins like fibrinogen, which enhances clot formation, and induces the expression of TF on monocytes, macrophages, and endothelial cells.^14,17^ Interestingly, NS1 has been reported to induce IL-6 during viral infection,^16^ supporting the participation of this cytokine in the hemostatic and inflammatory events that characterize flavivirus pathogenesis.

TF is the primary initiator of the extrinsic coagulation pathway and plays a pivotal role in thrombin generation and fibrin clot formation. TF binds to factor VII/VIIa, triggering the coagulation cascade, thrombin generation, and fibrin clot formation. It plays a crucial role in hemostasis, thrombosis, and inflammation. The IL-6-TF axis creates a prothrombotic state during inflammation, contributing to a dysregulated coagulation during severe flavivirus infections. Notably, the upregulation of TF by IL-6 also enhances platelet activation and promotes a feedback loop of inflammation and coagulation, which may worsen vascular damage reported during flavivirus infection.^18,19^

This study aims to investigate the differential effects of sNS1 proteins from various flaviviruses on the coagulation pathways. By comparing the coagulation-modulating activities of sNS1 derived from DENV, WNV, TBEV, JEV, and YFV, the study seeks to uncover how sNS1 contributes to virus-specific coagulopathy. Dissecting the virus-specific effects of sNS1 on coagulation, in conjunction with key host mediators like IL-6, may reveal new mechanisms of flavivirus pathogenesis and help identify targets for therapeutic intervention to mitigate vascular damage and coagulopathy.

## 2 Materials and Methods

### 2.1 Cell Lines and Treatment

THP-1 (ATCC TIB202) human monocyte cells were cultivated at 37 °C in a humidified atmosphere containing 5% CO_2_ in RPMI 1640 (Gibco), supplemented with 1% l-glutamine, 10% fetal bovine serum (FBS), and 1% penicillin/streptomycin. Cells were monitored 2–3 times per week for medium color changes and clump formation. Clumps were gently dispersed to obtain a single-cell suspension and split 1:1 with fresh medium, resulting in a cell density of 5–8 × 10^5^ cells/mL.

To assess Factor Xa generation (FXaG) experiments and avoid FBS interference, THP-1 cells were seeded in growth medium without FBS at 1 million per well in a 24-well plate and treated with different Flavivirus sNS1 for 24 h. All of the other experiments were performed in complete medium with 10% FBS.

### 2.2 sNS1 Treatments

sNS1 protein from eight different Flaviviruses (West Nile Virus – WNV, Dengue Virus 1-D1, Dengue Virus 2 – D2, Dengue Virus 3 – D3, Dengue Virus 4 – D4, Tick-Borne Encephalitis Virus – TBV, Yellow Fever Virus – YFV and Japanese Encephalitis Virus – JEV; FLAVX4-NS1, the Native Antigen, UK) were used at a final concentration of 5 μg/mL^14,20,21^ for 24 h. All recombinant sNS1 proteins were commercially obtained (The Native Antigen Company, UK), expressed in human HEK293 cells, with a declared purity of >99% verified by SDS-PAGE and HPLC. According to the manufacturer, the sNS1 proteins are predominantly in the biologically active hexameric form. Endotoxin contamination was below detectable levels (<0.05 EU/μg) as stated in the product certificates. The proteins were reconstituted and handled under sterile, endotoxin-free conditions according to the manufacturer’s recommendations.

LPS (from *E. coli* O111:B4, Sigma-Aldrich LPS25) 10 μg/mL was used as a positive control of THP1 activation, in terms of IL-6 and TF expression.

### 2.3 Viability Evaluation by MTT Assay

Cell viability was assessed by an MTT assay (Roche Diagnostics, Milan, Italy). Briefly, cells were treated for 24 h with the different sNS1 at a concentration of 5 μg/mL. Then, 10 μL of MTT solution was added to each well overnight. The following day, 100 μL of solvent was added, and after 4 h, the absorbance at 570 nm was measured. Treatment with DMSO 20% was used as a positive control of cell toxicity.^22^ THP-1 viability was also tested after 24 h in a FBS-free medium. Results are expressed as the mean value ± standard deviation (SD) of percent optical density (OD) derived from three independent experiments.

### 2.4 Factor Xa Generation (FXaG) Assay

The activated FXaG in human normal plasma from a minimum of 20 donors (Pool Norm, Diagnostica Stago Inc., USA) was triggered using 5 pM TF as an activator (Innovin, Dade Behring, Marburg, Germany) and evaluated by a specific FXa fluorogenic substrate (Spec-trafluor FXa, American Diagnostica, Stamford, CT, USA).^23^ We used a commercial pooled normal plasma to provide a standardized assay background and reduce interdonor variability during the comparative screening of multiple sNS1 proteins. Pooled plasma samples were diluted (1/20 final) in an HBS buffer (Hepes 20 mM, NaCl 150 mM, PEG-8000 0.1%, pH 7.4) and incubated for 45 min at 37 °C with media (1/10 final) from macrophages treated with different sNS1 proteins or sNS1 alone. The FXaG was initiated by the addition of a mixture of FXa substrate (110 μM), Innovin, and 5 mM CaCl_2_. The fluorescence was measured over time in a fluorimeter (excitation 355 nm, emission 460 nm, Fluoroskan Ascent BioMed, FL, USA). The reported range of FXaG parameters, which resulted from three independent experiments with all single assays performed in duplicate, varied from X to Y%, indicating the variability in the coagulation activation observed under different experimental conditions, in comparison to untreated samples. This range reflects the differences in the level of FXa generation, indicating an anticoagulant effect in the presence of ranges with negative-low values, or a procoagulant activity, when the range contains positive-higher percentages.

The commercial pooled normal plasma used was not specifically screened for antiflavivirus NS1 IgG. However, since pooled plasma derives from multiple donors, the contribution of any possible single donor with a high anti-NS1 titer is expected to be diluted.

### 2.5 ELISA Methods

THP-1 cell supernatants and purified microvesicle fractions were evaluated for IL-6 and TF by ELISA assays, using Human IL-6 ELISA Kit (Quantikine, Bio-Techne, MN, USA) and Human Tissue Factor Simple step ELISA Kit (Abcam, Cambridge, UK), respectively, following the manufacturer’s protocols. For the analysis of microvesicle fraction, 1–2 freeze–thaw cycles or a mild sonication (10–30 s) were performed before the assay, to guarantee proteins recover.

The presence of both IL-6 and TF in the samples was revealed by colorimetric reaction and read at 450 nm, with concentration determined by interpolation to the standard curve and reported as pg/mL. All samples were analyzed in triplicate, and data were reported as mean value ± SD.

### 2.6 TF Gene Expression Analysis

Gene expression analysis was performed on RNA extracted from THP-1 cells after the treatments described above using the PureLink RNA Mini kit (Invitrogen, Thermo Fisher, Milan, Italy). The extracted RNA was converted into cDNA using the RT High-Capacity Kit (Thermo Fisher, Milan, Italy). Gene expression analysis was performed by real-time quantitative PCR using PowerUp SYBR Green Master Mix (Thermo Fisher, Milan, Italy) and the following primer sets (PrimeTime, IDT, INC.): Hs.PT. 56a.27427814.g for TF and Hs.PT.39a.22214836 for GAPDH.

Amplification was performed as follows: 1 cycle at 50 °C for 2 min, 1 cycle at 95 °C for 2 min, and 40 cycles at 95 °C for 1 s and 60 °C for 30 s. Quantitative PCR analysis was performed using a QuantStudio3 real-time PCR detection system (Applied Biosystems, Thermo Fisher, Milan, Italy). Relative quantification of given mRNA levels for the samples was conducted using the 2^–ΔΔCT^ 2 (Delta–Delta CT) method,^24^ normalized to the constitutively expressed housekeeping gene GAPDH. Relative fold changes were generated comparing the noninfected control (NT) to the samples.

### 2.7 Microvesicle Isolation

Microvesicles (MVs) were isolated as previously described.^25^ THP-1 monocytes were treated with sNS1 for 24 h and then washed three times in PBS1x and once with Krebs-Ringer Solution (KRH). Cells were then resuspended in 1 mL of KRH and collected or treated with ATP 1 mM for 30 min at 37 °C as a positive control of MVs release. Then, cells were centrifuged twice at 880g for 5 min at 4 °C, keeping each time the supernatant, to remove cellular debris. Finally, the shedding vesicle fraction was obtained by centrifugation of the supernatant at 10,000*g* for 30 min at 4 °C. The pellet enriched in vesicles was collected in 150 L of KRH for flow cytometry analysis and ELISA assays.

### 2.8 Statistical Analysis

A paired Student’s *t-*test was used to perform a comparative analysis of individual parameters, relative gene expression normalized to GAPDH, and expressed as fold changes relative to the corresponding control group. Bonferroni correction was applied for multiple testing. *P*-values < 0.05 were considered statistically significant. Statistical analysis was conducted using GraphPad Prism version 10 software (GraphPad Prism v.10, La Jolla, CA, USA). All experiments were conducted in duplicate or triplicate, in three independent assays, and results are presented as mean ± standard deviation (SD).

## 3 Results

### 3.1 sNS1 Proteins Show Distinct Effects on Coagulation

We selected the THP-1 cell line to assess sNS1 and host–pathogen interactions because it closely replicates the cellular mechanisms underlying coagulation and inflammation in human monocytes, making it a suitable in vitro model for studying the interplay between viral proteins and the host system. Additionally, the standardized nature of the THP-1 cell line ensures reproducibility and consistency across experiments, which is essential for understanding how sNS1 contributes to the procoagulant and pro-inflammatory states associated with viral infections. The different sNS1 proteins were tested for their cytotoxic effects on THP-1 cells. The sNS1 were purchased from Native Antigen (UK). These sNS1 proteins are expressed in human HEK293 cells, ensuring proper folding and post-translational modifications that closely mimic the native viral antigens. The resultant proteins are predominantly in a hexameric conformation,^10^ which is considered the biologically active form involved in viral pathogenesis. This hexameric structure is achieved through hydrophobic interactions and lipid associations, forming a barrel-shaped complex. The treatment was performed for 24 h at a concentration of 5 μg/mL, as previously reported.^14,20,21^ As shown in Figure 1, none of the recombinant sNS1 tested affected the cell viability. On the contrary, treatment with DMSO 20% showed an almost total reduction of cell viability (Figure 1, *p* < 0.001, Student's *t-*test).

**Figure 1:**
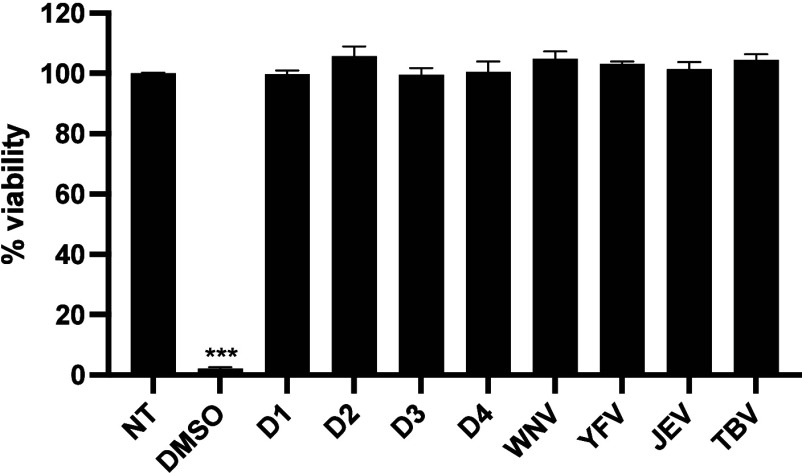
Cell viability evaluation by the MTT assay. Flavivirus sNS1 was added to THP-1 cells at a concentration of 5 μg/mL for 24 h. DMSO 20% was used as a positive control of cell toxicity. Data are reported as mean ± SD of three independent experiments. ***p < 0.001.

To evaluate the sNS1’s influence on TF-mediated coagulation, we evaluated the activation of factor X by an activated FXaG. TF is the primary initiator of the extrinsic coagulation pathway, forming a complex with Factor VIIa on the surface of cells or MVs. This TF-FVIIa complex then catalyzes the activation of Factor X to its active form, Factor Xa, which is essential for thrombin generation and subsequent fibrin clot formation.

Since monocytes are a key cell type involved in the production of TF, supernatants derived from the THP-1 monocytic cell line treated with the eight different sNS1 proteins for 24 h were tested. The treatments were conducted in an FBS-free medium to avoid interference with FXaG results. Deprivation of FBS for 24 h did not affect cell viability, even in the presence of stimulation with sNS1 (Suppl. Figure 1).

All FXaG assays were conducted in pooled plasma samples in the presence of a normal amount of coagulation trigger (TF 5 pM, from commercial thromboplastin Innovin), along with media from monocytes treated with sNS1 proteins (Figure 2A–D).

**Figure 2:**
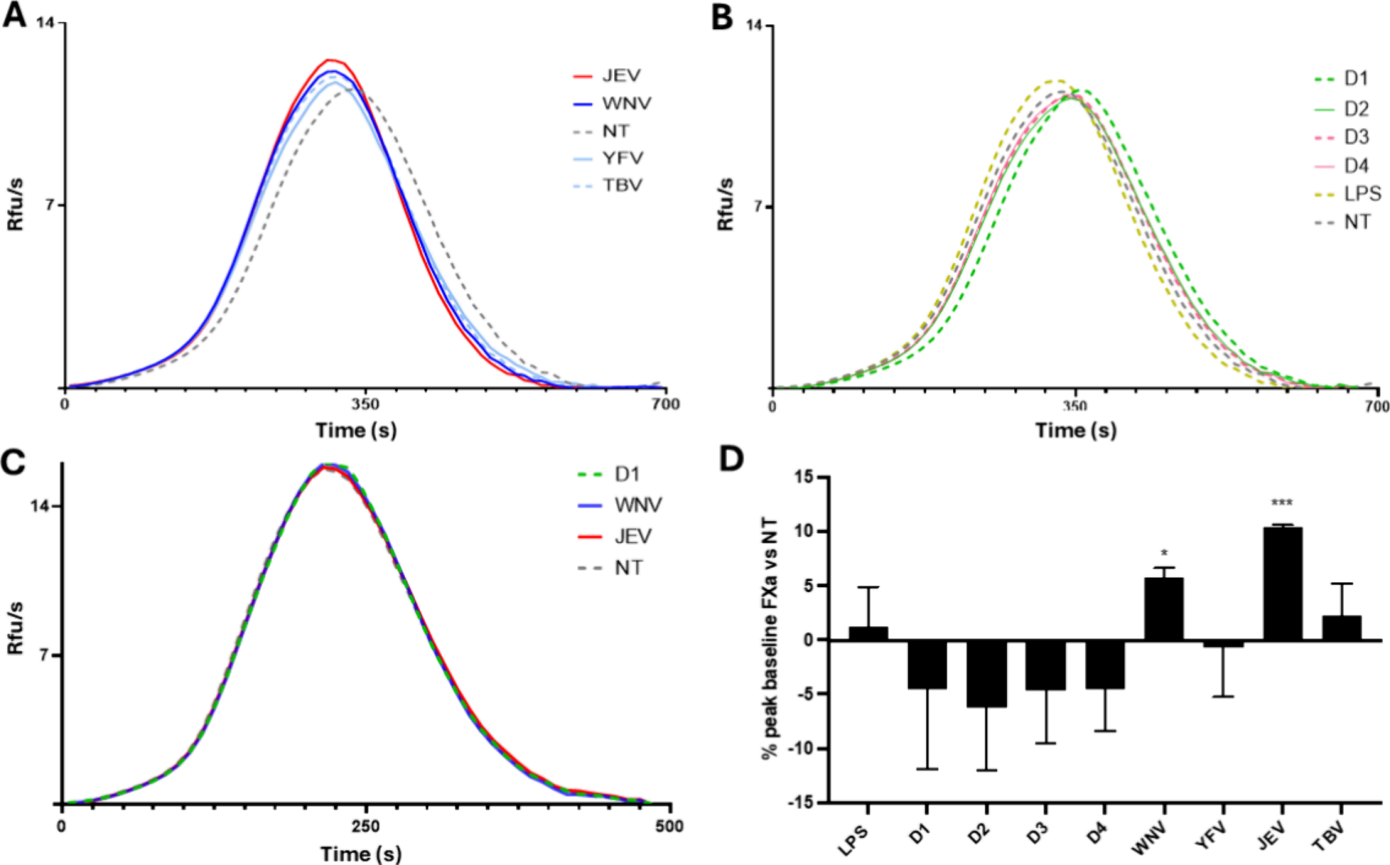
Factor Xa generations on infected media added to normal plasma. (A and B) FXaG in medium from cells treated or untreated with NS1 proteins (5ug/mL for 24 h) and triggered by 5 pM TF. FXa generation activity in commercial standardized pooled normal plasma (minimum 20 donors) added with medium from untreated cells (NT, dashed gray line), and treated cells with sNS1 from WNV (blue line), YFV (light-blue line), JEV (red line), TBV (dashed light-blue line), D1 (dashed green line), D2 (green line), D3 (dashed pink line), D4 (pink line). The stimulation with LPS (dashed yellow line) was used as a positive control. NT (dashed gray line) (C) FXaG triggered by 5 pM TF, in pooled normal plasma added with purified sNS1 proteins from D1 (dashed green line), WNV (blue line), JEV (red line), and NT (dashed gray line). All curves and samples of FXaG are virtually indistinguishable, indicating no direct activity in FXa generation of sNS1 proteins. (D) Peak baseline % compared to NT. Data were reported as SD ± mean of three replicates *p < 0.05, **p < 0.01, ***p < 0.001.

Figure 2 illustrates the time required for Factor X activation (“lag time,” measured in seconds). A rightward shift on the *x*-axis indicates a longer activation time and lower peaks reflecting an anticoagulant trend. Conversely, higher Rfu/s (Relative fluorescent units/second) values and peaks positioned earlier on the *x*-axis indicate a faster coagulation activation, signifying a procoagulant effect.

Treatment with various sNS1 proteins revealed contrasting effects. Specifically, sNS1 from neurotropic Flaviviruses, particularly WNV and JEV, demonstrated a procoagulant effect, as indicated by reduced lag time and increased peak of reaction, as compared with untreated cells (NT), as showed in Figure 2A,D (WNV from −2.1 to −14.8% of lag time, from 5.1 to 6.4% of peak, *p* < 0.05, Student’s *t-*test; YFV from −5.4 to −14.4% of lag time, from −3.9 to 2.6% of peak; JEV from −3.2 to −15.6% of lag time, from 10.3 to 10.6% of peak, *p* < 0.001, Student’s *t-*test, and TBV, from −7.8 to −18.7% of lag time, from 0.1% to 4.4% of peak). The different effect on coagulation observed in the presence of treatment with sNS1 from the neurotropic flaviviruses could be ascribable to differences in amino acid composition, glycosylation, and oligomer assembly of sNS1 from TBV, WNV, YFV, and JEV.^10,26–28^ In fact, these variations could alter sNS1 binding affinity to host cell surfaces and influence their capacity to trigger cellular responses, as previously described concerning endothelial permeability.^14,20^

In contrast, sNS1 from hemorrhagic flaviviruses (D1–4) induced an anticoagulant effect, evidenced by prolonged lag times and reduced reaction peaks as compared to NT (Figure 2B,D; D1 from 8.1 to 20.6% of lag time, from 0.7 to −9.8% of peak; D2 from −0.3 to 7.4% of lag time, from −2.2 to −10.4% of peak; D3 from 1.4 to 8.5% of lag time, from −1.1 to −8.1% of peak; D4 from 8.2 to 10.2% of lag time, from −1.9 to −7.3% of peak). sNS1 from D1 prolonged the FXaG lag time more than all other sNS1 proteins tested, making it the most anticoagulant sNS1 molecule in our experimental setting. Thus, neurotropic sNS1 proteins (WNV, JEV, YFV, and TBV) consistently induced a procoagulant pattern in this model, while hemorrhagic sNS1 proteins (DENV1–4) exhibited an anticoagulant tendency. These findings confirm that WNV and JEV sNS1 predominantly elicit a procoagulant, not anticoagulant, effect. This pattern supports the concept that localized procoagulant activation in monocytes or microglia-like cells may contribute to endothelial activation and immune cell recruitment during neurotropic flavivirus infection.

To exclude a direct effect of sNS1 on the coagulation cascade, we selected sNS1 proteins from D1, WNV, and JEV for individual testing in the FXaG assay. These three sNS1 proteins were chosen because of the different effects observed (Figure 2A,B). As shown in Figure 2C, none of the sNS1 added to pooled plasma samples significantly differed from untreated plasma in terms of FXaG, excluding a direct effect of sNS1 proteins on the assay.

Furthermore, the absence of any direct effect of purified sNS1 proteins on FXaG (Figure 2C) supports a model in which the observed modulation of coagulation is mediated by cell-derived factors rather than direct sNS1–plasma interactions. This demonstrates that the contribution of any possible single donor of the pooled plasma with a high anti-NS1 titer is expected to be diluted, not affecting the result.

Based on the FXaG screening across eight sNS1 proteins (Figure 2), we selected three representative sNS1 proteins (WNV and JEV as a prototypical neurotropic/procoagulant; D1 as a prototypical hemorrhagic/anticoagulant molecule) for detailed TF mRNA and protein analyses, in order to maximize mechanistic contrast while limiting the scope of in-depth assays.

### 3.2 TF Evaluation

Since sNS1 from neurotropic flaviviruses, particularly WNV and JEV, demonstrated a procoagulant effect, we evaluated the direct effect on TF expression, as TF serves as the primary initiator of the extrinsic coagulation cascade, leading to thrombin generation and fibrin clot formation.^29^

We investigated the effect of sNS1 from WNV, JEV, and D1 on TF mRNA expression (Figure 3A). Gene expression analysis revealed that sNS1 from neurotropic flaviviruses WNV and JEV induced a higher TF mRNA transcription (Figure 3, *p* < 0.001 and *p* < 0.01 for sNS1 of WNV and JEV, respectively, Student’s *t*-test), compared to sNS1 from D1 (Figure 3, *p* = ns, Student’s *t*-test). This result was also confirmed by the analysis of cell supernatants for TF amount by the ELISA assay. In fact, sNS1 from WNV and JEV induced an increase of TF amount (Figure 3B *p* < 0.001, Student’s *t*-test), compared to the basal condition, while sNS1 from D1 showed a lower induction (Figure 3B *p* < 0.05, Student’s *t*-test).

**Figure 3:**
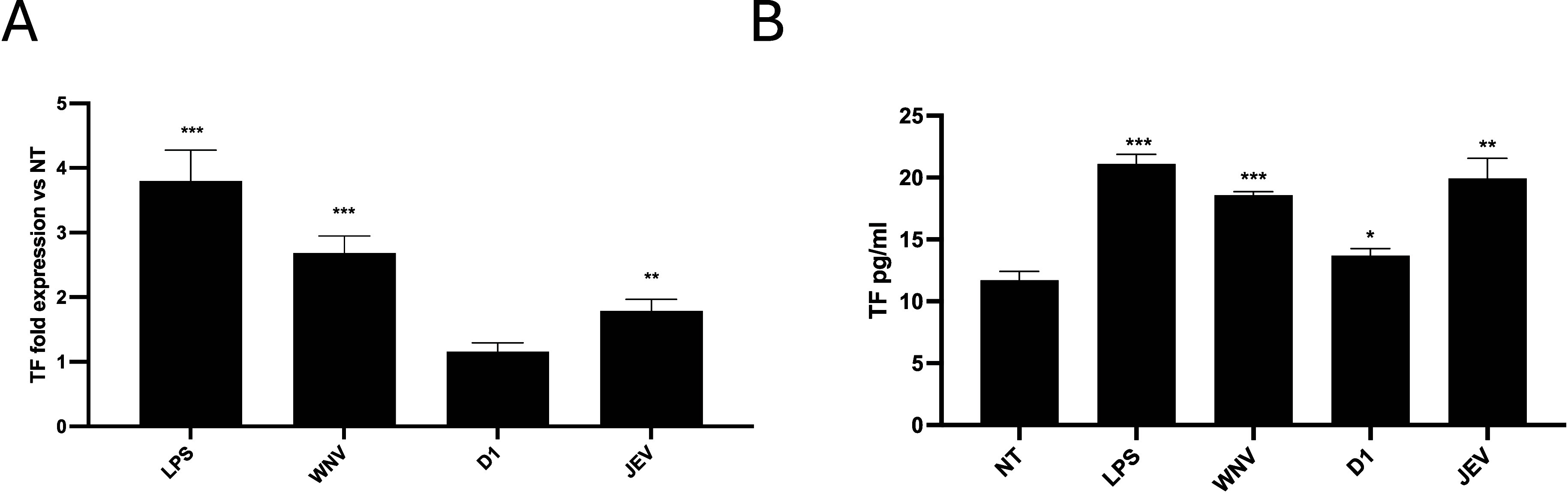
Evaluation of the TF expression. Flavivirus sNS1 was added to THP-1 cells at a concentration of 5 μg/mL for 24 h. The stimulation with lipopolysaccharides (LPS) was used as a positive control. NT: untreated samples. (A) Gene expression analysis was performed by RT-qPCR, and data are reported as fold change vs NT. (B) TF content in cell supernatants was evaluated by an ELISA assay. Data are reported as SD ± mean of three replicates. *p < 0.05, **p < 0.01, ***p < 0.001.

### 3.3 Evaluation of MV Release and TF Content

It is known that TF’s procoagulant effect is functional to its association with membranes.^30^ TF is a transmembrane glycoprotein that serves as the primary initiator of the extrinsic coagulation cascade by binding to Factor VII. Besides its role as a membrane-bound protein, a significant amount of TF can be expressed on the surface of secreted MVs, triggering coagulation activation at distant sites.^31,32^ To determine if the differential effects observed on FX generation might involve TF carried by MV, THP-1 supernatants were analyzed for the MV content and TF membrane expression.

To enhance MV yield and improve the sensitivity of TF detection, we treated THP-1 cells with sNS1 in the presence of ATP, a known inducer of MV shedding.^33,34^ ATP stimulation promotes MV release via purinergic signaling, thus increasing the amount of vesicular material available for downstream analysis. This strategy allowed us to better evaluate the association of TF with MVs in response to different sNS1 proteins.

Figure 4 shows representative plots and histograms for MV identification. ATP treatment was used as a positive control for MV release.^33,34^ We first assessed THP-1 cells for MV release and reported an increased amount of MVs in the supernatant after stimulation with ATP (Figure 4, *p* < 0,01 and *p* < 0.5, respectively, Student’s *t*-test), confirming the ability of this cell line to shed MVs.

**Figure 4:**
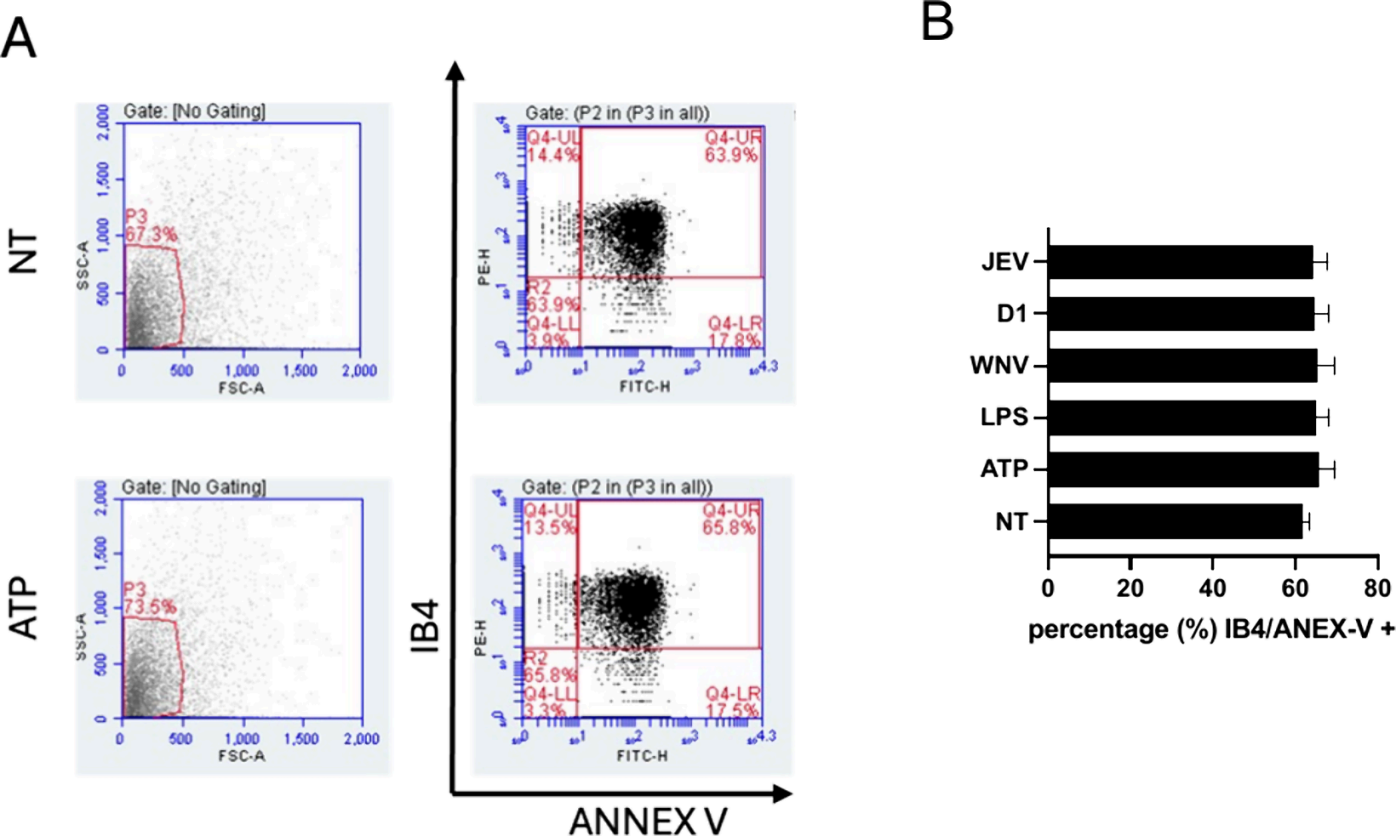
Evaluation of MV release. THP-1 cells were stimulated with ATP (1 mM), and MV release was evaluated. (A) Representative flow cytometry dot plots and histograms showing gating strategy for MV identification, based on size (side-scatter-area, SSC-A- vs forward-scatter-area, FSC-A), specific MVs markers – IB4 and Annexin-V (ANNEX-V) for untreated (NT) and ATP-stimulated samples (ATP); (B) percentage of MVs identified as IB4/ANNEV-V+ of total events gated. Data are reported as SD ± mean of three replicates.

Moreover, we confirmed that the gating strategy adopted, based on cell complexity and size (SSC vs FSC) and IB4 and Annexin-V expression (Figure 4A),^35^ correctly identified MVs (Figure 4A).

Then, ATP-stimulated THP-1 were treated with the different sNS1 for 24 h. The addition of the different sNS1 did not alter the induction of MVs released (Figure 4B), suggesting a possible difference in the MVs TF content.

To assess this hypothesis, the purified MV fractions were analyzed for TF loading by an ELISA assay.

As shown in Figure 5, sNS1 from neurotropic flaviviruses exhibits an increase in TF amount carried by MVs compared to untreated samples (Figure 5, WNV and JEV vs NT, *p* < 0.001, Student’s *t*-test), while no significant difference was observed in the presence of treatment with D1 sNS1.

**Figure 5:**
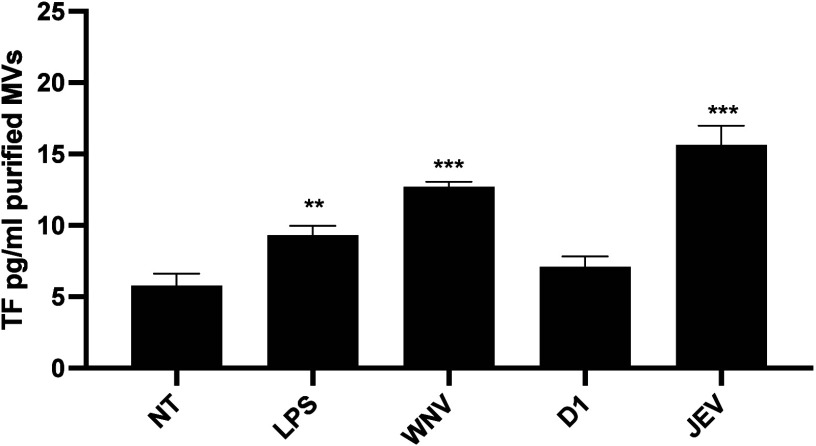
Evaluation of the TF amount in purified MV fractions. THP-1 cells were stimulated with ATP (1 mM), and flavivirus sNS1 was added at a concentration of 5 μg/mL for 24 h. The stimulation with lipopolysaccharides (LPS) was used as a positive control. NT: untreated samples. TF content in purified MV fraction was evaluated by ELISA assay. Data are reported as SD ± mean of three replicates. *p < 0.05, **p < 0.01, ***p < 0.001.

These results confirm the ability of sNS1 to differentially affect the association of TF with MVs. The differential amount of TF loaded in MVs reported in THP-1 supernatants after treatment with sNS1 is consistent with the coagulant and inflammatory effects observed in the presence of the different sNS1.

### 3.4 sNS1 Proteins' Effect on the Inflammation Cascade

We hypothesize that the observed MV release may involve soluble sNS1 binding to specific cellular receptors (e.g., toll-like receptors),^36^ triggering intracellular signaling pathways that lead to the upregulation of inflammatory mediators, as pro-inflammatory cytokines, and membrane remodeling enzymes.^37^ Additionally, sNS1-induced alterations in the lipid bilayer could enhance membrane shedding,^38^ facilitating the formation of MVs. Pro-inflammatory cytokines, such as interleukin-6 (IL-6), play a pivotal role in inducing coagulation by creating a prothrombotic environment. IL-6 stimulates the expression of TF on the surface of endothelial cells, monocytes, and macrophages, which serves as the primary initiator of the coagulation cascade.^39^

Elevated IL-6 levels can amplify TF expression, contributing to a hypercoagulable state that is commonly observed in severe flavivirus infections.^40,41^ Moreover, significantly higher levels of MVs and IL-6 were reported to associate with coagulopathy due to viral infections, as observed in COVID-19 patients.^42^ Based on this, we examined the influence of sNS1 proteins on IL-6 secretion by monocytes as a marker of inflammatory induction.

Our results showed that sNS1 proteins derived from neurotropic flaviviruses significantly induced IL-6 secretion compared to untreated cells (NT) (Figure 6A, WNV vs NT and JEV vs NT, *p* < 0.01, Student’s *t-*test). In contrast, sNS1 proteins from D1, which previously exhibited anticoagulant effects and a reduced release of TF carried by MVs, did not induce IL-6 secretion (Figure 6A, D1 vs NT, *p* = ns, Student’s *t-*test), with levels remaining comparable to baseline.

**Figure 6:**
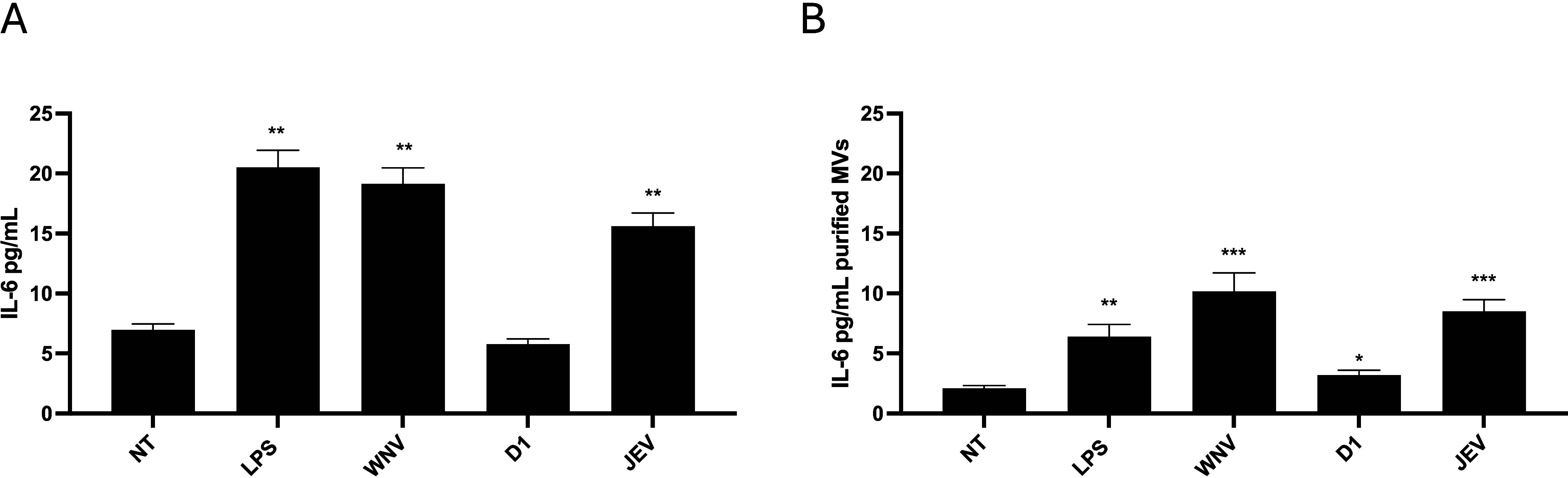
Evaluation of IL-6 release. IL-6 concentration was evaluated in THP-1 cells treated with flavivirus sNS1, which was added to THP-1 cells at a concentration of 5 μg/mL for 24 h. The stimulation with lipopolysaccharides (LPS) was used as a positive control. NT: untreated samples. (A) IL-6 levels in cell supernatants and (B) in the MVs purified fraction, after ATP stimulation. Data are reported as SD ± mean of three replicates. *p < 0.05, **p < 0.01, ***p < 0.001.

Notably, IL-6 evaluation in purified MV fractions after sNS1 stimulation showed similar results (Figure 6B, WNV and JEV vs NT, *p* < 0.001; D1 vs NT, *p* = 0.05, Student’s *t-*test), supporting the interconnection among higher IL-6 and TF levels observed during treatment with sNS1 from neurotropic flaviviruses, and their role in procoagulant effect.

## 4 Discussion

This pilot study provides comparative in vitro evidence that different flavivirus sNS1 proteins differentially modulate monocyte TF expression, TF-bearing MV content, and IL-6 secretion, suggesting cell-specific pathways by which sNS1 may contribute to virus-specific alterations in hemostasis. Although we did not include blocking or inhibition experiments in this pilot study, the correlated increase in TF expression, TF-bearing MVs, and IL-6 secretion observed after neurotropic sNS1 treatment supports a monocyte-dependent, sNS1-driven procoagulant axis. Future mechanistic work, including blocking or neutralization studies (e.g., anti-sNS1, anti-TF, or anti-IL-6 antibodies), will be essential to confirm causality. Our findings highlight the virus-specific modulation of hemostasis, where hemorrhagic flaviviruses (e.g., DENV) predominantly induce anticoagulant effects, while neurotropic flaviviruses (e.g., WNV and JEV) promote a hypercoagulable state (Figure 2). This phenomenon appears to be mediated, at least in part, by the differential impact of sNS1 on TF expression (Figure 3) and its release within MVs (Figure 5). These results build upon earlier studies demonstrating the pivotal role of NS1 in pathogenesis and its interactions with endothelial and immune cells, as previously described.^35^

The apparent discrepancy between the procoagulant effects observed with WNV and JEV sNS1 and the clinical hemorrhagic phenotype more often associated with DENV can be reconciled by considering the cell-type and organ specificity of sNS1 actions. Dengue NS1 has been shown to exert potent effects on endothelial cells, platelets, and hepatocytes,^43^ leading to glycocalyx disruption, platelet activation, and impaired hepatic synthesis of coagulation factors^44^—mechanisms that promote bleeding through barrier failure and consumption of clotting factors. In contrast, our THP-1 monocyte model selectively highlights the effects of sNS1 on monocyte TF induction and TF-bearing MVs. In this cellular context, neurotropic sNS1 (WNV/JEV) elicits greater TF/MV responses than DENV sNS1. Therefore, rather than contradicting clinical observations, our results suggest that different flavivirus sNS1 proteins interact with distinct host cell compartments to produce divergent systemic outcomes. This pattern suggests that DENV sNS1 interacts differently with monocytes compared with neurotropic sNS1 proteins. While WNV and JEV sNS1 directly stimulate IL-6 and TF expression, DENV sNS1 may act mainly on endothelial or hepatic cells, leading to consumption coagulopathy and vascular leakage instead of TF-mediated hypercoagulation. In the case of neurotropic viruses such as WNV and JEV, localized monocyte activation and TF induction may promote endothelial activation and vascular inflammation within neural tissues, potentially facilitating viral persistence and immune evasion. Specifically, given the functional and ontogenetic similarities between monocytes and microglia, it is plausible that the effects observed in monocytes may also apply to microglia, supporting a role in CNS-related pathogenesis.

The distinct modulation of MVs content by sNS1 proteins aligns with the previously proposed mechanisms during viral infections,^45,46^ in which MVs were shown to amplify the inflammatory and coagulation cascade. Here, we demonstrate that neurotropic sNS1 proteins enhance the TF content in MVs (Figure 5), underscoring the complexity of flavivirus-host interactions. The functional relevance of increased TF is supported by two observations: (i) TF is specifically enriched in purified MVs from WNV/JEV sNS1-treated cells (Figure 5), consistent with membrane-localized, procoagulant TF, and (ii) conditioned media from these cells shorten FXaG lag time and increase peak intensity (Figure 2). The absence of any direct effect of purified sNS1 on FXaG (Figure 2C) further supports a cell-mediated MV-dependent mechanism. Importantly, no direct FXa activation was detected upon incubation of purified sNS1 proteins with plasma (Figure 2C), indicating that the procoagulant activity depends on sNS1-induced host cell responses—likely mediated by TF-bearing MVs and IL-6 release.

The role of MVs as vectors for coagulation signals corroborates previous findings in similar viral contexts.^20,47^

The ability of neurotropic sNS1 proteins to trigger the release of MVs and promote TF expression may be explained by their activation of inflammatory pathways that amplify coagulation regulation. The ability of the NS1 protein to affect the coagulation cascade has also been recently described in liver and platelet cells,^44,48^ supporting our findings.

As a proof of concept, we observed an increase in IL-6 levels induced by neurotropic sNS1 proteins in both cell supernatants and purified MVs (Figure 6). These findings point to a possible regulatory role of IL-6 in TF expression, establishing a link among sNS1 proteins, inflammatory signaling, and the coagulation modulation observed during flavivirus infections.

The structural and sequence differences in flavivirus sNS1 proteins that contribute to their distinct effects on coagulation stem from variations in amino acid residues, glycosylation patterns, and surface-exposed domains.^49^ These differences influence how sNS1 interacts with host cells, coagulation factors, and immune components.^50^ Specific sequence differences in the β-ladder and wing domains of sNS1 alter its ability to interact with endothelial cells, platelets, and coagulation proteins.^27,51^ Hemorrhagic flaviviruses, such as DENV, often have residues that disrupt endothelial barrier function, leading to vascular leakage and an anticoagulant environment.^14^ In contrast, neurotropic flaviviruses such as WNV have sequence motifs that enhance the binding of sNS1 to monocytes and endothelial cells, promoting TF expression. Variations in N-linked glycosylation sites between flaviviruses influence sNS1’s stability and receptor interactions. For instance, the glycan structures on sNS1 from neurotropic viruses may enhance its ability to induce TF expression and support the formation of TF-enriched MVs, whereas hemorrhagic viruses might exhibit glycan patterns that dampen these procoagulant pathways. Differences in the oligomeric forms of sNS1 (dimer vs hexamer) affect its functionality.^52^ Hemorrhagic flaviviruses tend to produce sNS1 structures with a higher propensity for endothelial disruption, while neurotropic flaviviruses produce sNS1 structures that efficiently interact with coagulation factors like Factor X and facilitate MV formation. Structural differences in lipid-binding regions may influence how sNS1 interacts with cell membranes^53^ and promotes MV release.

Previous studies have demonstrated that flavivirus NS1 proteins can disrupt endothelial integrity and activate immune signaling (e.g., TLR4-dependent activation),^11^ with tissue specificity reflecting disease tropism. Our data extend this framework by showing differential induction of monocyte TF and TF-bearing MVs by neurotropic versus hemorrhagic sNS1 proteins, suggesting that monocyte-derived procoagulant signals may be an additional mechanism contributing to sNS1-mediated pathogenesis in neurotropic infections.

These findings are summarized in Figure 7, which illustrates the proposed model: neurotropic sNS1 proteins engage monocytes to upregulate IL-6 and TF, promoting release of TF-bearing MVs that enhance Factor Xa activation (procoagulant profile), whereas hemorrhagic sNS1 proteins display limited activation and correlate with anticoagulant outcomes.

**Figure 7:**
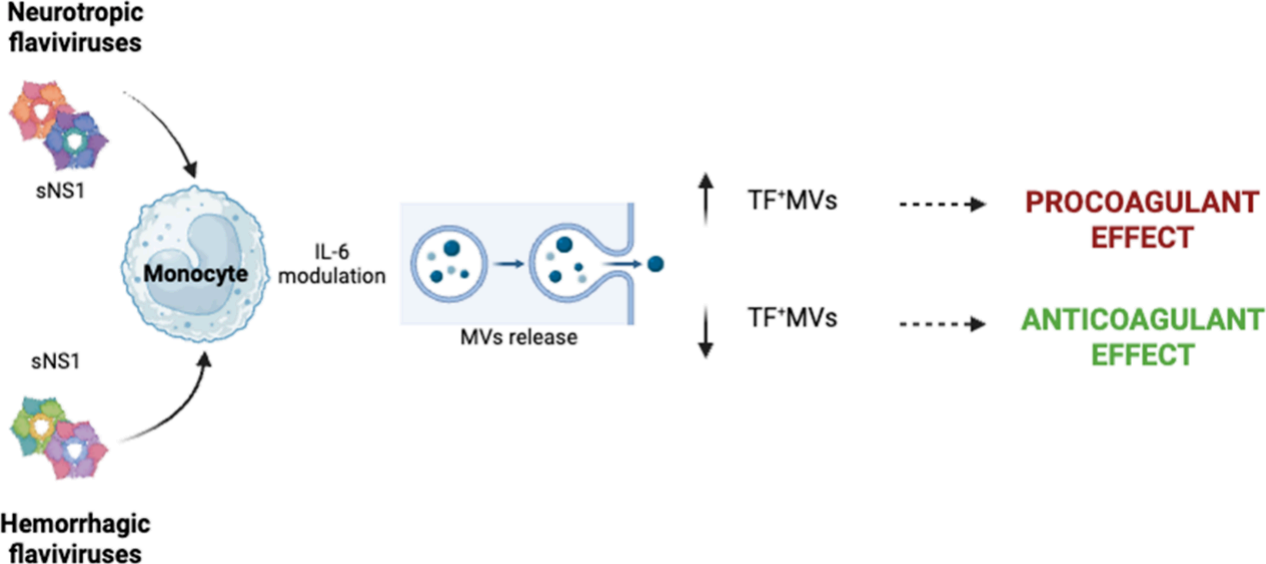
Proposed model of the sNS1-driven modulation of coagulation in THP-1 cells. Schematic representation of how neurotropic flavivirus sNS1 induces IL-6 and TF expression, leading to the release of TF-bearing MVs (TF^+^NVs) and increased procoagulant, whereas hemorrhagic sNS1 proteins show reduced activation, corresponding to an anticoagulant profile.

Further analysis will be essential to identify and evaluate the specific components contributing to the observed differences in coagulation effects induced by sNS1 proteins of different flaviviruses. This includes dissecting the roles of key structural features, such as amino acid sequence variations, glycosylation patterns, and lipid-binding domains, in mediating interactions with host coagulation pathways. Advanced biochemical, structural, and functional assays are required to determine how these variations influence TF induction, Factor X activation, and MV formation. Additionally, studies focusing on host-specific factors, such as receptor binding affinities, cellular signaling pathways, and the influence of the immune response, will provide a more comprehensive understanding of the mechanisms at play.

By combining molecular dynamics simulations, mutational analyses, and in vivo models, future research can uncover the precise contributions of these components, facilitating the development of targeted therapeutic interventions to mitigate flavivirus-induced coagulopathy.

Moreover, analysis of sera from infected patients (TF, MV levels, IL-6, and anti-NS1 serology) would be a high-priority follow-up to establish clinical correlation. We encourage future studies to examine well-phenotyped clinical cohorts to determine whether the monocyte/MV-TF pathway we observe in vitro is reflected in patient plasma and is associated with clinical outcomes.

These insights will not only point out the molecular basis of flavivirus-specific pathogenesis but also might suggest potential therapeutic strategies. For instance, targeting TF-bearing MVs or modulating IL-6 signaling to regulate TF expression may represent a promising approach in the treatment of neurotropic flaviviruses infections.^54^

## 5 Limitations

A key limitation of our study is that while we observed a consistent correlation between sNS1-induced IL-6 secretion and TF expression, including TF-bearing MVs, we did not directly demonstrate causality through blocking or inhibition assays. While our data suggest a correlation between sNS1-induced IL-6 secretion and increased TF levels, particularly in TF-bearing MVs, we did not directly demonstrate a causal relationship. The phenotypic differences among sNS1 proteins and the lack of direct effects in plasma suggest an sNS1/monocyte/MV-TF pathway; however, definitive mechanistic studies, such as neutralization or blocking experiments (using anti-sNS1, anti-IL-6/IL-6R, or anti-TF antibodies), are needed to prove causality and identify therapeutic targets. Since our goal was to conduct a comparative screening of flavivirus sNS1 proteins rather than dissect specific mechanisms, blocking assays were not included in this initial study. Nevertheless, the correlated upregulation of TF, TF-bearing MVs, and IL-6 in response to neurotropic sNS1 supports the presence of a cell-mediated sNS1–IL-6–TF/MV axis, which should be explored in future mechanistic investigations.

We did not measure anti-NS1 antibodies in the pooled plasma used for FXaG. Although the direct incubation of purified sNS1 with pooled plasma did not affect FXaG (Figure 2C), we cannot fully exclude a modulatory role of low-titer anti-NS1 antibodies present in donor plasma. Future studies should include testing individual patient plasmas and screening for anti-NS1 IgG to definitively exclude this possibility. Again, the use of pooled plasma reduces biological variability but does not capture interhost differences; future work should address individual donor responses and correlate them with anti-NS1 serostatus.

Lastly, as our findings are based on an in vitro monocytic model (THP-1 cells), we were ordered to exploit reproducibility for TF induction assays and their widespread use as a surrogate for human monocytes. Nonetheless, THP-1 cells do not capture the full phenotype of primary monocytes or other cell types implicated in coagulation (endothelium, platelets, and hepatocytes). Validation of our findings in primary human monocytes, ex vivo patient samples, and in vivo models will be necessary to assess physiological relevance.

Nonetheless, the consistency of these results across multiple sNS1 proteins supports the existence of a cell-mediated sNS1–IL-6–TF/MV axis, which warrants further experimental validation.

## 6 Conclusions

In conclusion, this study explores the nuanced role of flavivirus sNS1 proteins in modulating coagulation and inflammation, delineating the distinct impacts of hemorrhagic versus neurotropic flavivirus sNS1 on TF-bearing MVs release and IL-6 production, aiming to increase our understanding of flavivirus pathogenesis. Moreover, these virus-induced alterations in hemostasis, driven by sNS1, may create a microenvironment that facilitates viral replication by enhancing immune evasion, increasing endothelial permeability, and modulating host inflammatory responses, ensuring efficient virus dissemination and persistence. Importantly, by defining an sNS1–IL-6–TF/MV axis in monocytes, our findings establish a mechanistic link between viral proteins and dysregulated host responses that may serve as a basis for therapeutic intervention. Approaches such as targeting IL-6 signaling, inhibiting TF activity, or neutralizing sNS1—already under exploration in other inflammatory coagulopathies—could represent promising strategies to mitigate vascular injury and hypercoagulability in severe flavivirus infections. These insights, corroborated by a growing body of literature, emphasize the importance of host–pathogen dynamics in determining clinical outcomes. Future research should prioritize the development of targeted therapies aimed at mitigating the pathogenic effects of sNS1 and its downstream mediators, particularly in severe flavivirus infections. This work lays the groundwork for novel approaches to address the challenges posed by these globally significant pathogens.

## Supplementary Material



## Notes

Supplementary Figure 1, Cell viability evaluation by the MTT assay (PDF)

## References

[1] Zhao R., Wang M., Cao J., Shen J., Zhou X., Wang D., Cao J. (2021). flavivirus: From Structure to Therapeutics
Development. Life (Basel).

[2] Danis-Lozano R., Diaz-Gonzalez E. E., Malo-Garcia I. R., Rodriguez M. H., Ramos-Castaneda J., Juarez-Palma L., Ramos C., Lopez-Ordonez T., Mosso-Gonzalez C., Fernandez-Salas I. (2019). Vertical transmission of dengue virus
in Aedes aegypti and its role in the epidemiological persistence of
dengue in Central and Southern Mexico. Trop
Med. Int. Health.

[3] Griffiths M. J., Turtle L., Solomon T. (2014). Japanese encephalitis
virus infection. Handb. Clin. Neurol..

[4] Waggoner J. J., Rojas A., Pinsky B. A. (2018). Yellow
Fever Virus: Diagnostics for
a Persistent Arboviral Threat. J. Clin. Microbiol..

[5] Wang W. H., Urbina A. N., Chang M. R., Assavalapsakul W., Lu P. L., Chen Y. H., Wang S. F. (2020). Dengue
hemorrhagic
fever - A systemic literature review of current perspectives on pathogenesis,
prevention and control. J. Microbiol Immunol
Infect.

[6] Muller D. A., Young P. R. (2013). The flavivirus NS1 protein: molecular
and structural
biology, immunology, role in pathogenesis and application as a diagnostic
biomarker. Antiviral Res..

[7] Lin S. W., Chuang Y. C., Lin Y. S., Lei H. Y., Liu H. S., Yeh T. M. (2012). Dengue virus nonstructural
protein NS1 binds to prothrombin/thrombin
and inhibits prothrombin activation. J. Infect.

[8] Bhatt P., Sabeena S. P., Varma M., Arunkumar G. (2021). Current Understanding
of the Pathogenesis of Dengue Virus Infection. Curr. Microbiol..

[9] Edeling M. A., Diamond M. S., Fremont D. H. (2014). Structural basis
of flavivirus NS1
assembly and antibody recognition. Proc. Natl.
Acad. Sci. U. S. A..

[10] Pan Q., Jiao H., Zhang W., Chen Q., Zhang G., Yu J., Zhao W., Hu H. (2024). The step-by-step assembly mechanism
of secreted flavivirus NS1 tetramer and hexamer captured at atomic
resolution. Sci. Adv..

[11] Modhiran N., Watterson D., Muller D. A., Panetta A. K., Sester D. P., Liu L., Hume D. A., Stacey K. J., Young P. R. (2015). Dengue virus NS1
protein activates cells via Toll-like receptor 4 and disrupts endothelial
cell monolayer integrity. Sci. Transl. Med..

[12] Beatty P. R., Puerta-Guardo H., Killingbeck S. S., Glasner D. R., Hopkins K., Harris E. (2015). Dengue virus
NS1 triggers endothelial permeability
and vascular leak that is prevented by NS1 vaccination. Sci. Transl. Med..

[13] Beltrami S., Rizzo S., Schiuma G., Cianci G., Narducci M., Baroni M., Di Luca D., Rizzo R., Bortolotti D. (2025). West Nile
virus non-structural protein 1 promotes amyloid Beta deposition and
neurodegeneration. Int. J. Biol. Macromol..

[14] Puerta-Guardo H., Glasner D. R., Espinosa D. A., Biering S. B., Patana M., Ratnasiri K., Wang C., Beatty P. R., Harris E. (2019). flavivirus
NS1 Triggers Tissue-Specific Vascular Endothelial Dysfunction Reflecting
Disease Tropism. Cell Rep.

[15] Shwetank, Date O. S., Kim K. S., Manjunath R. (2013). Infection
of human endothelial cells by Japanese encephalitis
virus: increased expression and release of soluble HLA-E. PLoS One.

[16] Rastogi M., Sharma N., Singh S. K. (2016). flavivirus NS1:
a multifaceted enigmatic
viral protein. Virol. J..

[17] Tenno T., Botling J., Oberg F., Nilsson K., Siegbahn A. (1997). Tissue factor
expression in human monocytic cell lines. Thromb
Res..

[18] Heidari Z., Naeimzadeh Y., Fallahi J., Savardashtaki A., Razban V., Khajeh S. (2024). The Role of Tissue Factor In Signaling
Pathways of Pathological Conditions and Angiogenesis. Curr. Mol. Med..

[19] Auroni T. T., Arora K., Natekar J. P., Pathak H., Elsharkawy A., Kumar M. (2023). The critical role of
interleukin-6 in protection against neurotropic
flavivirus infection. Front. Cell Infect. Microbiol..

[20] Puerta-Guardo H., Biering S. B., de Sousa F. T. G., Shu J., Glasner D. R., Li J., Blanc S. F., Beatty P. R., Harris E. (2022). flavivirus NS1 Triggers
Tissue-Specific Disassembly of Intercellular Junctions Leading to
Barrier Dysfunction and Vascular Leak in a GSK-3beta-Dependent Manner. Pathogens.

[21] Puerta-Guardo H., Glasner D. R., Harris E. (2016). Dengue Virus
NS1 Disrupts the Endothelial
Glycocalyx, Leading to Hyperpermeability. PLoS
Pathog.

[22] de
Abreu Costa L., Henrique Fernandes Ottoni M., Dos Santos M. G., Meireles A. B., Gomes de Almeida V., de Fatima Pereira W., Alves de Avelar-Freitas B., Eustaquio Alvim Brito-Melo G. (2017). Dimethyl Sulfoxide
(DMSO) Decreases Cell Proliferation and TNF-alpha, IFN-gamma, and
IL-2 Cytokines Production in Cultures of Peripheral Blood Lymphocytes. Molecules.

[23] Baroni M., Martinelli N., Lunghi B., Marchetti G., Castagna A., Stefanoni F., Pinotti M., Woodhams B., Olivieri O., Bernardi F. (2020). Aptamer-modified
FXa generation assays
to investigate hypercoagulability in plasma from patients with ischemic
heart disease. Thromb Res..

[24] Livak K. J., Schmittgen T. D. (2001). Analysis
of relative gene expression data using real-time
quantitative PCR and the 2­(-Delta Delta C­(T)) Method. Methods.

[25] Bianco F., Pravettoni E., Colombo A., Schenk U., Möller T., Matteoli M., Verderio C. (2005). Astrocyte-derived ATP induces vesicle
shedding and IL-1 beta release from microglia. J. Immunol.

[26] Avirutnan P., Hauhart R. E., Somnuke P., Blom A. M., Diamond M. S., Atkinson J. P. (2011). Binding of flavivirus nonstructural protein NS1 to
C4b binding protein modulates complement activation. J. Immunol.

[27] Biering S. B., Akey D. L., Wong M. P., Brown W. C., Lo N. T. N., Puerta-Guardo H., Tramontini Gomes de Sousa F., Wang C., Konwerski J. R., Espinosa D. A., Bockhaus N. J., Glasner D. R., Li J., Blanc S. F., Juan E. Y., Elledge S. J., Mina M. J., Beatty P. R., Smith J. L., Harris E. (2021). Structural basis for antibody inhibition of flavivirus
NS1-triggered endothelial dysfunction. Science.

[28] Xu X., Song H., Qi J., Liu Y., Wang H., Su C., Shi Y., Gao G. F. (2016). Contribution of intertwined loop
to membrane association revealed by Zika virus full-length NS1 structure. EMBO J..

[29] Mackman N. (2004). Role of tissue
factor in hemostasis, thrombosis, and vascular development. Arterioscler Thromb Vasc Biol..

[30] Rao L. V., Kothari H., Pendurthi U. R. (2012). Tissue
factor: mechanisms of decryption. Front. Biosci.
(Elite Ed.).

[31] Wu C., Lu W., Zhang Y., Zhang G., Shi X., Hisada Y., Grover S. P., Zhang X., Li L., Xiang B., Shi J., Li X. A., Daugherty A., Smyth S. S., Kirchhofer D., Shiroishi T., Shao F., Mackman N., Wei Y., Li Z. (2019). Inflammasome Activation Triggers Blood Clotting and Host Death through
Pyroptosis. Immunity.

[32] He Y., Wu Q. (2023). The Effect of Extracellular
Vesicles on Thrombosis. J. Cardiovasc Transl
Res..

[33] Blonda M., Amoruso A., Grasso R., Di Francescantonio V., Avolio C. (2017). Multiple Sclerosis Treatments Affect Monocyte-Derived
Microvesicle Production. Front Neurol.

[34] Colombo F., Bastoni M., Nigro A., Podini P., Finardi A., Casella G., Ramesh M., Farina C., Verderio C., Furlan R. (2018). Cytokines Stimulate the Release of
Microvesicles from
Myeloid Cells Independently from the P2 × 7 Receptor/Acid Sphingomyelinase
Pathway. Front. Immunol..

[35] Bianco F., Perrotta C., Novellino L., Francolini M., Riganti L., Menna E., Saglietti L., Schuchman E. H., Furlan R., Clementi E., Matteoli M., Verderio C. (2009). Acid Sphingomyelinase activity triggers microparticle
release from glial cells. EMBO J..

[36] Modhiran N., Watterson D., Blumenthal A., Baxter A. G., Young P. R., Stacey K. J. (2017). Dengue virus NS1 protein activates immune cells via
TLR4 but not TLR2 or TLR6. Immunol Cell Biol..

[37] Rasmussen N. S., Nielsen C. T., Jacobsen S., Nielsen C. H. (2019). Stimulation of Mononuclear
Cells Through Toll-Like Receptor 9 Induces Release of Microvesicles
Expressing Double-Stranded DNA and Galectin 3-Binding Protein in an
Interferon-alpha-Dependent Manner. Front. Immunol..

[38] Ci Y., Yang Y., Xu C., Qin C. F., Shi L. (2021). Electrostatic
Interaction Between NS1 and Negatively Charged Lipids Contributes
to flavivirus Replication Organelles Formation. Front Microbiol.

[39] Chin B. S., Conway D. S., Chung N. A., Blann A. D., Gibbs C. R., Lip G. Y. (2003). Interleukin-6, tissue factor and
von Willebrand factor
in acute decompensated heart failure: relationship to treatment and
prognosis. Blood Coagul Fibrinolysis.

[40] Neumann F. J., Ott I., Marx N., Luther T., Kenngott S., Gawaz M., Kotzsch M., Schomig A. (1997). Effect of human recombinant interleukin-6
and interleukin-8 on monocyte procoagulant activity. Arterioscler Thromb Vasc Biol..

[41] Szotowski B., Antoniak S., Poller W., Schultheiss H. P., Rauch U. (2005). Procoagulant soluble tissue factor
is released from endothelial cells
in response to inflammatory cytokines. Circ.
Res..

[42] Tayer A. H., Jahromi H. K., Kamravan M., Farhangdoost F., Ahmadi T., Kolaei M. (2024). Evaluation of circulating
microvesicles
and their procoagulant activity in patients with COVID-19. BMC Res. Notes.

[43] Malavige G. N., Ogg G. S. (2017). Pathogenesis of
vascular leak in dengue virus infection. Immunology.

[44] Das S., Mallik M. H., Chattopadyay P., Mallick S., Karmakar D., Ghora S., Begum F., Chatterjee B., Thagriki D. S., Srivastava A. K., Ray U. (2024). Dengue virus NS1 leads
to downregulation of HNF4 alpha in liver cells resulting in a decrease
in coagulation factors I, V, X, and XIII, contributing to coagulopathy. J. Virol..

[45] El
Safadi D., Lebeau G., Lagrave A., Mélade J., Grondin L., Rosanaly S., Begue F., Hoareau M., Veeren B., Roche M., Hoarau J.-j., Meilhac O., Mavingui P., Despres P., Viranaicken W., Krejbich-Trotot P. (2022). Extracellular Vesicles are Conveyors of the NS1 Toxin
during flavivirus Infection. Viruses.

[46] Zhang S., He Y., Wu Z., Wang M., Jia R., Zhu D., Liu M., Zhao X., Yang Q., Wu Y., Zhang S., Huang J., Ou X., Gao Q., Sun D., Zhang L., Yu Y., Chen S., Cheng A. (2023). Secretory
pathways and multiple functions of nonstructural protein 1 in flavivirus
infection. Front. Immunol..

[47] Martinez-Rojas P. P., Monroy-Martinez V., Agredano-Moreno L. T., Jimenez-Garcia L. F., Ruiz-Ordaz B. H. (2024). Zika Virus-Infected Monocyte Exosomes
Mediate Cell-to-Cell
Viral Transmission. Cells.

[48] Quirino-Teixeira A. C., Rozini S. V., Barbosa-Lima G., Coelho D. R., Carneiro P. H., Mohana-Borges R., Bozza P. T., Hottz E. D. (2020). Inflammatory signaling
in dengue-infected platelets requires translation and secretion of
nonstructural protein 1. Blood Adv..

[49] Carpio K. L., Barrett A. D. T. (2021). flavivirus NS1
and Its Potential in Vaccine Development. Vaccines
(Basel).

[50] Tan B. E. K., Beard M. R., Eyre N. S. (2023). Identification
of Key Residues in
Dengue Virus NS1 Protein That Are Essential for Its Secretion. Viruses.

[51] Gogokhia L., Buhrke K., Bell R., Hoffman B., Brown D. G., Hanke-Gogokhia C., Ajami N. J., Wong M. C., Ghazaryan A., Valentine J. F., Porter N., Martens E., O’Connell R., Jacob V., Scherl E., Crawford C., Stephens W. Z., Casjens S. R., Longman R. S., Round J. L. (2019). Expansion of Bacteriophages
Is Linked to Aggravated Intestinal Inflammation and Colitis. Cell Host Microbe.

[52] Latanova A., Starodubova E., Karpov V. (2022). Flaviviridae Nonstructural
Proteins:
The Role in Molecular Mechanisms of Triggering Inflammation. Viruses.

[53] Kim S. Y., Li B., Linhardt R. J. (2017). Pathogenesis
and Inhibition of Flaviviruses from a
Carbohydrate Perspective. Pharmaceuticals (Basel).

[54] Zeng Q., Liu J., Hao C., Zhang B., Zhang H. (2023). Making sense of flavivirus
non-strctural protein 1 in innate immune evasion and inducing tissue-specific
damage. Virus Res..

