# Supplementary material for: Flavivirus Nonstructural Protein 1‑Driven Coagulation via Tissue Factor-Bearing Microvesicles: A Pilot Study
Source: ACS Omega. 2025 Nov 14;10(46):56645–55. doi: 10.1021/acsomega.5c09129 (PMC12658609; doi:10.1021/acsomega.5c09129)
Supplement: Supplementary file 1 [file ao5c09129_si_001.pdf]

Flavivirus NS1–Driven Coagulation via TF-Bearing Microvesicles: a Pilot Study

Silvia Beltrami 1, §, Matteo Ferraresi 1, §, Giorgia Cianci 1, Marco Narducci 1, 2, Roberta Rizzo 1,, Marcello Baroni 3,§ and Daria Bortolotti 1, §, **

1 Department of Environmental and Prevention Sciences, University of Ferrara, Ferrara, Italy; silvia.beltrami@unife.it; giorgia.cianci@unife.it; matteo.ferraresi@edu.unife.it; rbr@unife.it; brtdra@unife.it

2 Temple University, Japan Campus, Tokyo, Japan; marco.narducci@tuj.temple.edu

3 Department of Life Sciences and Biotechnology, University of Ferrara, Ferrara, Italy; marcello.baroni@unife.it

* *Correspondence: rbr@unife.it; Tel.+39 0523455382, brtdra@unife.it, Tel.+39 0523455398*

§ *Equally contributed*

Keywords: Flavivirus; NS1; Coagulation, Inflammation, Microvesicles, Tissue-factor.

SUPPORTING INFORMATION FOR PUBLICATION

THP-1 viability Cell viability was evaluated in the absence of FBS (no FBS) during Flavivirus sNS1 treatment at the concentration of 5 µg/mL for 24 hours

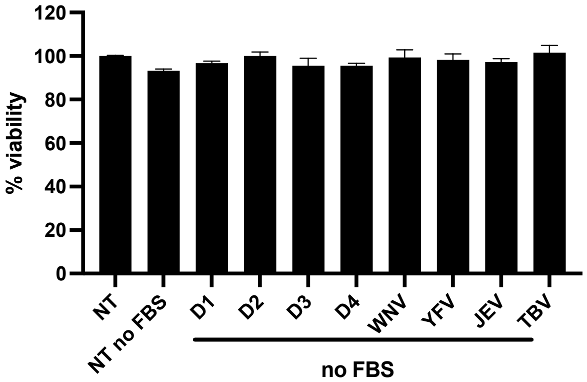

Supplementary Figure 1. Cell viability evaluation by MTT assay. THP-1 viability was evaluated in the absence of FBS (no FBS) during flavivirus sNS1 treatment at the concentration of 5 µg/mL for 24 hours, in comparison with untreated cells (NT) grown in the presence of FBS, considered as 100% of cell viability. Data are reported as mean ± SD of three independent experiments.
